# Structural dynamics of the two-component response regulator RstA in recognition of promoter DNA element

**DOI:** 10.1093/nar/gku572

**Published:** 2014-07-02

**Authors:** Yi-Chuan Li, Chung-ke Chang, Chi-Fon Chang, Ya-Hsin Cheng, Pei-Ju Fang, Tsunai Yu, Sheng-Chia Chen, Yi-Ching Li, Chwan-Deng Hsiao, Tai-huang Huang

**Affiliations:** 1 Institute of Molecular Biology, Academia Sinica, Taipei 115, Taiwan, ROC; 2 Institute of Bioinformatics and Structural Biology, National Tsing Hua University, Hsinchu 300, Taiwan; 3 Institute of Biomedical Sciences, Academia Sinica, Taipei 115, Taiwan, ROC; 4 Genomics Research Center, Academia Sinica, Taipei 115, Taiwan, ROC; 5 Department of Physics, National Taiwan Normal University, Taipei 116, Taiwan, ROC

## Abstract

The RstA/RstB system is a bacterial two-component regulatory system consisting of the membrane sensor, RstB and its cognate response regulator (RR) RstA. The RstA of Klebsiella pneumoniae (kpRstA) consists of an N-terminal receiver domain (RD, residues 1–119) and a C-terminal DNA-binding domain (DBD, residues 130–236). Phosphorylation of kpRstA induces dimerization, which allows two kpRstA DBDs to bind to a tandem repeat, called the RstA box, and regulate the expression of downstream genes. Here we report the solution and crystal structures of the free kpRstA RD, DBD and DBD/RstA box DNA complex. The structure of the kpRstA DBD/RstA box complex suggests that the two protomers interact with the RstA box in an asymmetric fashion. Equilibrium binding studies further reveal that the two protomers within the kpRstA dimer bind to the RstA box in a sequential manner. Taken together, our results suggest a binding model where dimerization of the kpRstA RDs provides the platform to allow the first kpRstA DBD protomer to anchor protein–DNA interaction, whereas the second protomer plays a key role in ensuring correct recognition of the RstA box.

## INTRODUCTION

Bacteria and plants frequently use two-component signal transduction systems (TCSs) to adapt to environmental changes and to survive under stress conditions (1–4). TCSs are especially abundant in bacteria and absent in metazoans, making them attractive targets for antibacterial drug discovery (5). Typical TCSs couple a transmembrane histidine protein kinase (HK), which detects changes in the environment, to a cytosolic response regulator (RR), which often alters gene expression. External stimulus leads to autophosphorylation of the HK, which is then able to form a complex with the cognate RR and allow the transfer of the phosphate group to a conserved Asp residue of the RR. Phosphorylation alters intra-molecular interactions within the RR, which allows it to bind to DNA, resulting in up- or down-regulation of downstream genes (6).

The RstB/RstA system is a ubiquitous TCS composed of the membrane-associated histidine kinase RstB and its cognate response regulator RstA. In *Escherichia coli* and *Salmonella*, RstA is under the control of the PhoP/PhoQ TCS, which monitors extracellular Mg^2+^ levels, and up-regulates the acid-induced *asr* (acid shock RNA) gene and the biofilm regulator *csgD* gene under acidic conditions (2,7,8). Overexpression of RstA in *Salmonella enterica* serovar Typhimurium induced degradation of RpoS and altered biofilm formation (9). Moreover, RstA directly binds to the promoter of the *feo* operon in *Salmonella* and promotes the expression of the iron transporter FeoB under iron-replete conditions (10). Since both biofilm formation and iron acquistion are often associated with the ability of the organism to establish infection (11,12), RstA may play a regulatory role in virulence. Preliminary experiments on *Yersinia pseudotuberculosis* have also shown that mutations on the *rstA* gene attenuated virulence in mouse models (13).

*Klebsiella pneumoniae* is an important pathogen associated with nosocomial infections (14). Emergence of *K. pneumoniae* variants with multiple antimicrobial resistances has become a serious problem in hospitals worldwide. Association of RstB/RstA with multiple virulence factors highlights its potential as a drug target, and emphasizes the importance of investigating its mechanism of action. *K. pneumoniae* RstA (*kp*RstA) contains an N-terminal receiver domain (RD, residues 1–119) and a C-terminal DNA-binding domain (DBD, residues 131–236), with an overall sequence identity of 79% compared to RstA from *E. coli* and *S. enterica* serovar Typhimurium (see Supplemental Figure S1). As a member of the OmpR/PhoB subfamily, phosphorylation of the conserved Asp in the RD is believed to result in dimerization of the domain through a conserved α4–β5–α5 region, accompanied by the binding of the DBD to cognate sequences in the promoter region of RstA-regulated genes. Genomic systematic evolution of ligands by exponential enrichment (SELEX) experiments in *E. coli* have shown that RstA binds to the consensus sequence TACATNTNGTTACA with tandem TACA recognition sites (8). Like other members of the OmpR/PhoB subfamily, it is implied that dimerization of RstA is necessary for binding to this tandem sequence, with each DBD binding to a single recognition site (15,16). The consensus sequence, called the RstA box, can also accommodate imperfections in the TACA sequence. *Klebsiella* RstA is capable of activating the RstA box of the *asr* gene in *K. pneumoniae*, which contains a RstA box with a TACA/TACT imperfect repeat instead of the canonical TACA repeat found in the *E. coli asr* gene (17).

Although proteins of the OmpR/PhoB subfamily share a high degree of structural similarity, there is little consensus on how they recognize the tandem DNA sequences in the context of a dimer. For example, the two DBDs of the OmpR dimer can bind to different DNA sequences in either head-to-tail or head-to-head orientations (18–20), whereas the DBDs of PhoB and PmrA bind to DNA exclusively in a head-to-tail orientation (21,22). Moreover, binding of PhoB to DNA induces bending in the DNA, which is not observed for PmrA. These observations imply divergent structural bases for tandem sequence recognition within the same protein subfamily. Here we report the characterization of *kp*RstA binding to the RstA box sequence located in the *asr* promoter of *K. pneumoniae* by isothermal titration calorimetry (ITC). The structures of *kp*RstA RD activated with BeF_3_^−^, free *kp*RstA DBD and *kp*RstA DBD in complex with DNA are also disclosed through a combination of X-ray crystallography and nuclear magnetic resonance (NMR) spectroscopy. Furthermore, we compare the backbone dynamics of the DBD in the bound and free forms. Surprisingly, the *kp*RstA dimer binds to the RstA box DNA sequence in a sequential fashion which has never been observed in other RRs. The combination of multifaceted approaches allows us to formulate a sequence of events behind the activation and unique DNA recognition mechanism of *kp*RstA.

## MATERIALS AND METHODS

### Chemicals and reagents

BeSO_4_ was purchased from Mitsuwas Pure Chemicals (Japan). Other chemical reagents were purchased from Sigma (USA) and Merck (USA). DNA sequences were synthesized by Genomics Ltd (Taiwan). Double-stranded DNA was obtained by mixing together complementary strands in the buffer for the current experiment, followed by denaturation at 95°C for 5 min and slowly cooling down to room temperature.

### Cloning, protein expression and purification

Full-length *kp*RstA was cloned into a modified pET32a(+) vector which lacked the region coding for the enterokinase cleavage site. *kp*RstA RD and *kp*RstA DBD were cloned into pET6H vector (a gift from Prof. J.-J. Lin, National Yang Ming University, Taiwan). The proteins were expressed in *E. coli* BL21(DE3) strain by growing the cells in 1.0 liter LB medium at 37°C until reaching an OD_600_ of 0.6–0.8 followed by induction with 1.0 mM isopropyl β-d-1-thiogalactopyranoside (IPTG). The cells were then allowed to grow overnight at 18°C before harvest. Isotope-labeled proteins for NMR experiments were produced by centrifuging and re-suspending the cells in 250 ml 2xM9 medium supplemented with 1.0 g/l of ^15^NH_4_Cl and/or 1.0 g/l u-^13^C-glucose prior to induction. Deuterated samples were obtained by substitution of water and u-^13^C-glucose with ^2^H_2_O and u-^13^C,^2^H-glucose in this step. To solve the crystallographic phase problem, selenomethionine (SeMet)-labeled samples of *kp*RstA RD and *kp*RstA DBD were obtained by growing *E. coli* B834(DE3) strain in LeMaster medium supplemented with 50 μg/ml SeMet, 1% glucose, 1 mM MgSO_4_, 0.25 mg/ml MgSO_4_ and 7.68 μg/ml FeSO_4_ during the induction phase. To achieve sufficient labeling, we constructed the *kp*RstA DBD(L153M/L168M) mutant where both Leu153 and Leu168 were changed to Met. Another loss-of-function mutant, R207A, was constructed for ITC experiments. The mutations were introduced into pET6H-*kp*RstA-DBD through site-directed mutagenesis by polymerase chain reaction (PCR) with the primers listed in Supplemental Table S1. All expressed proteins contained an N-terminal His-tag that was later removed in the case of full-length *kp*RstA.

The cells were lysed with a microfluidizer in loading buffer (20 mM Tris, 100 mM NaCl, pH 7.5) and the lysate spun down at 30 000 *g* on an Avanti J-26XP centrifuge (Beckman-Coulter, USA). The supernatant was loaded onto Ni-NTA resin (Qiagen, USA) pre-equilibrated with the loading buffer and eluted with the same buffer containing 300 mM imidazole. The eluted proteins were further purified by size-exclusion chromatography using an Akta fast-performance chromatography system (FPLC) equipped with a HiLoad Superdex-75 16/60 column (GE Healthcare, USA) equilibrated with the elution buffer solution (20 mM sodium phosphate, 50 mM NaCl, pH 6.0). The His-tag of full-length *kp*RstA was removed by digestion with 75 U of thrombin (GE Healthcare, USA) for 2 days at 4°C and the cleaved protein was further purified following manufacturer's instructions. The resulting full-length *kp*RstA contained a GSAMA sequence at the N-terminus, whereas both *kp*RstA-RD and *kp*RstA-DBD contained an extraneous N-terminal M-6H-AMG sequence.

### Isothermal titration calorimetry

ITC was performed with a Microcal ITC_200_ calorimeter (GE Healthcare, USA) at 25°C. Protein and DNA solution were dialyzed overnight against the same reaction buffer (20mM Tris, pH7.5, 100 mM NaCl in the case of *kp*RstA DBD and 20mM Tris, pH7.5, 100 mM NaCl, 7 mM MgCl_2_ with or without 5.3 mM BeSO_4_ and 35 mM NaF for full-length *kp*RstA). For *kp*RstA DBD, the titration was conducted by injecting 1 μl (first injection) or 2 μl (second to 19th injections) of 2 mM protein solution into 200 μl of 80 μM DNA (16- or 22-bp) solution. For full-length *kp*RstA, the titration was conducted by injecting 1.2 μl (first to 17th injections) or 1.6 μl (18th to 29th injections) of 1.0 mM protein solution into 200 μl of 57 μM 22-bp DNA solution. An initial delay of 180 s was applied before the first injection, with a 120 s interval between two successive injections. The binding isotherms of *kp*RstA DBD against 16-bp DNA were fitted to a single-site binding model while those of *kp*RstA DBD and full-length *kp*RstA against the 22-bp DNA were fitted to a sequential binding model under the Microcal Origin package (GE Healthcare, USA).

### NMR experiments

The NMR spectra were acquired on Bruker AVANCE 500, 600 or 800 MHz spectrometers (Bruker, Germany) equipped with 5-mm triple resonance cryoprobes at 25 or 37°C. NMR data were acquired in Shigemi tubes on 0.1–1.0 mM protein samples in NMR buffer (20 mM sodium phosphate, 50 mM NaCl, 1 mM EDTA, 1 mM DTT, 10% (v/v) D_2_O) at pH 6 for free *kp*RstA DBD and at pH 6.5 for the *kp*RstA DBD/DNA complex. The ^15^N,^13^C- and ^15^N,^13^C,^2^H-labeled protein–-DNA complex samples were prepared at a protein-to-DNA molar ratio of 1:1.2. As the 16-bp DNA substrate gave the best NMR spectra, they were used for all NMR studies. Protein backbone resonance assignments of *kp*RstA DBD and its complex with DNA were achieved by standard triple resonance experiments, including HNCA, HN(CO)CA, HNCACB, CBCA(CO)NH, HNCO and HN(CA)CO (23–27). Ambiguities in the backbone assignment were resolved by specific amino labeling of the protein. Complete assignment of free *kp*RstA DBD has been described elsewhere (28). ^1^H chemical shifts were externally referenced to 0 ppm through the methyl resonance of 2,2-dimethyl-2-silapentane-5-sulfonate (DSS), whereas ^13^C and ^15^N chemical shifts were indirectly referenced according to the recommendations of the International Union of Pure and Applied Chemistry (29). The weighted chemical shift perturbations (CSPs) of *kp*RstA DBD backbone ^15^N and ^1^H resonances upon DNA binding were calculated with the following equation: Δ*δ* = [(Δ*δ*_HN_)^2 ^+ (0.154Δ*δ*_N_)^2^]^1/2^.

Both ^13^C- and ^15^N-edited NOESY-HSQC experiments were conducted with a mixing time of 120 ms to obtain distance restraints for the structure calculation of free *kp*RstA DBD. The NMR spectra were processed using Bruker TOPSPIN 3.0 and analyzed with Sparky (Goddard, T.D. and Kneller, D.G., University of California San Francisco) and CARA (available from: http://www.nmr.ch). To measure one-bond ^1^H-^15^N residual dipolar couplings (RDC), free *kp*RstA DBD was partially aligned in liquid crystalline phase containing 15 mg/ml Pf1 bacteriophage (Asla Biotech Ltd, Latvia). Changes in splitting relative to the isotropic ^1^J_NH_ values were measured using DSSE–HSQC experiments to obtain one bond ^1^H-^15^N RDCs (30). The measured RDCs were analyzed using the program PALES (31).

The ^15^N spin-lattice/longitudinal relaxation rate, ^15^N-R_1_, spin–spin/transverse relaxation rate, ^15^N-R_2_, and [^1^H-^15^N]NOE were determined by using standard pulse sequences (32). Each ^15^N-R1 was determined with ten randomly ordered delays of 0.8, 118.8, 238.8, 358.8, 478.8, 598.8, 718.8, 898.8, 1018.8 and 1288.8 ms. Similarly, each ^15^N-R2 was determined from 10 delays, in random order, of 0, 34.3, 51.5, 68.6, 102.9, 120.1, 154.4, 188.7, 205.8 and 223.0 ms for free *kp*RstA DBD; 0, 17.2, 34.3, 51.5, 68.6, 85.8 and 102.9 ms for the *kp*RstA DBD/DNA complex. Both rate constants were determined using the program Protein Dynamics Center (Bruker, Germany), assuming mono-exponential decay of the peak intensities. The errors in peak intensities were calculated from two duplicate experiments. The steady-state heteronuclear [^1^H–^15^N] NOE experiment was carried out in duplicate in an interleaved manner, with and without proton saturation. The NOE was calculated as the error-weighed average ratio of peak intensities, with error estimated by standard deviation of three pairs of repeated experiments. The reduced spectral density analysis was performed as previously described (33–37).

### NMR structure determination

The solution structure of *kp*RstA DBD was calculated with CYANA 3.0 based on experimental restraints (38,39). Distance restraints were generated by picking and quantifying NOE peaks using the automatic peak picking routine of AURELIA (Bruker, Germany) followed by automated NOE cross-peak assignment with CYANA 3.0. The restraints were grouped into short- (≤1 Å), middle- (1–5 Å) and long-range (≥5 Å) distances. Backbone torsion angle restraints were predicted from backbone chemical shifts with TALOS+ (40), and only those identified as ‘good’ by the program were used in the structure calculation. Hydrogen bond restraints were generated for secondary structure regions by assigning the following distance constraints: 1.8–2.6 Å for H^N^–O and 2.7–3.5 Å for N–O. A list of restraint statistics is provided in Supplemental Table S2. Measured RDCs (71 out of 119) were also used in the calculation. Out of 150 calculated conformers, the 20 conformers with the lowest target functions were deposited to the Protein Data Bank (PDB) with accession code: 2MLK. All structural figures were prepared with MOLMOL (41) and PyMOL (42).

### Crystallization and data collection

For crystallization trials, *kp*RstA DBD was mixed with 23-bp DNA substrate (Figure 2C) at a molar ratio of 2:1 (DNA:protein) and concentrated to 20 mg/ml in preparation buffer (20 mM Tris-HCl, pH 7.5 and 100 mM NaCl). *kp*RstA RD was similarly prepared except that the preparation buffer contained additional 5.3 mM BeSO_4_, 35 mM NaF and 7 mM MgCl_2_. Initial crystallization trials were performed with commercially available kits (Hampton Research, USA) using the hanging drop vapor diffusion method (43). Specifically, 1 μl of concentrated complex was mixed with an equal amount of reservoir solution and equilibrated against 500 μl of reservoir solution at 26°C. After optimization of crystallization conditions, rhombus-shaped *kp*RstA RD crystals appeared in 100 mM Bis–Tris, pH 6.5 and 25% (v/v) polyethylene glycol 3350, whereas *kp*RstA DBD/DNA formed plate-shaped crystals in 100 mM Bis–Tris, pH 5.5, 28% (v/v) polyethylene glycol 400 and 1.32 M sodium formate. The phase of *kp*RstA RD crystals were determined by single-wavelength anomalous diffraction (SAD) of SeMet-labeled crystals at 0.97874 Å (Se-peak). The phase of *kp*RstA DBD/DNA crystals were determined by collecting multi-wavelength anomalous diffraction (MAD) data on a single SeMet-labeled crystal of *kp*RstA DBD(L153M/L168M)–DNA complex at three different wavelengths: 0.97879 Å (Se-edge), 0.96357 Å (Se-remote) and 0.97862 Å (Se-peak). All data were collected on an ADSC Quantum-315 CCD area detector at beamline BL13B1 of the National Synchrotron Radiation Research Center, Hsinchu, Taiwan. X-ray diffraction data integration and scaling were performed using the HKL2000 package (44). For the MAD experiment, data from each wavelength were indexed according to the same crystal orientation matrix, but integrated and scaled independently. Diffraction data statistics are listed in Supplemental Table S2.

**Figure 1.**
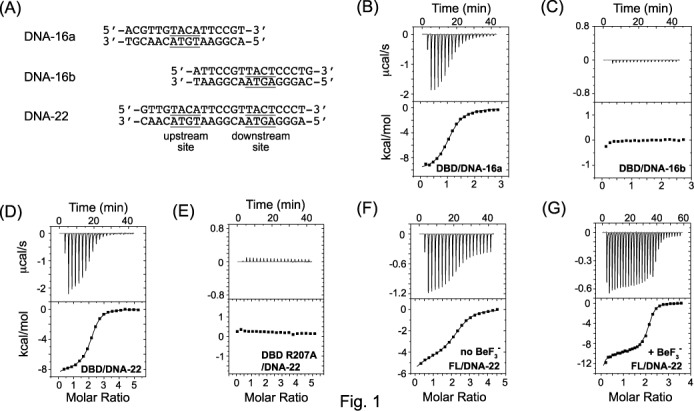
Determination of binding isotherm of *kp*RstA DBD with various RstA box DNA by isothermal titration calorimetry. The sequences of the duplex DNA used in this study are listed in (**A**). Binding of *kp*RstA DBD to DNA-16a (**B**) and DNA-16b (**C**) or DNA-22 (**D**) are shown with the ITC traces in the upper panel and the binding isotherms in the lower panel. The binding isotherm of the *kp*RstA DBD R207A mutant to DNA-16a is shown in (**E**). Binding of full-length *kp*RstA to DNA-22 is shown in (**F**) and that of *kp*RstA in the presence of BeF_3_^−^ is shown in (**G**). Detailed fitting parameters are listed in Table 1.

**Figure 2.**
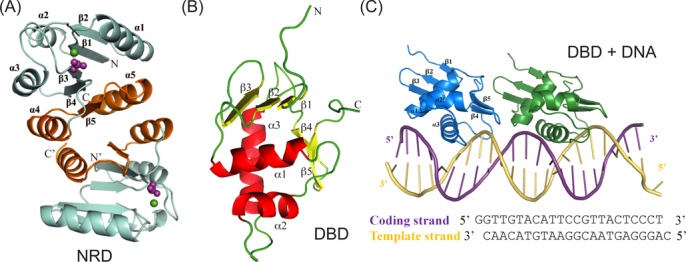
Structural overview of *kp*RstA domains. (**A**) Crystal structure of the *kp*RstA RD dimer in the presence of BeF_3_^−^. Dimer formation is mediated by the α4-β5-α5 interfaces highlighted in gold. The ligands within the phosphorylation site are shown as spheres with Mg^2+^ in green and BeF^3-^ in magenta. (**B**) Solution structure of *kp*RstA DBD with the helices and β-strands highlighted in red and yellow, respectively. (**C**) Crystal structure of *kp*RstA DBD in complex with DNA-23, which contains the same sequence as DNA-22 with overhanging G bases to facilitate crystallization. Each DNA-23 binds to two DBD molecules, with the upstream and downstream protomers colored in blue and green, respectively.

### Crystal structure determination and refinement

Crystals of *kp*RstA RD and *kp*RstA DBD/DNA complex diffracted to 3.1 and 2.7 Å, respectively. Experimental phases of *kp*RstA DBD/DNA complex were obtained at 2.7 Å using the MAD method implemented in the program AutoSol from the PHENIX suite (45). The programs COOT (46) and PHENIX were then used for rounds of manual model rebuilding and refinement, respectively. The final model for *kp*RstA DBD/DNA complex contained two proteins (102 residues in chain A and 101 residues in chain B), one double-stranded DNA molecule (22 nucleotides in chain C and 23 nucleotides in chain D) and 63 water molecules in the asymmetric unit. The structure was refined to a final R_work_ and R_free_ of 21.6 and 27.1%, respectively, with good stereochemistry. The structure of *kp*RstA RD was solved by employing the MR-SAD protocol as implemented in PHENIX (47) using a poly-alanine monomer of the PhoB RD structure (PDB code: 1ZES) as the search model. Molecular replacement was performed with the AutoMR program within PHENIX using the data set collected at the absorption edge (0.97891 Å). The resulting model with one dimer in the asymmetric unit was used for initial refinement, model phase calculations, location of selenium positions, SAD phasing, phase combination, density modification and model building in an automatic manner. The final model contained four *kp*RstA RD molecules (corresponding to two dimers, with 117 residues in each chain), four BeF_3_^−^, four Mg^2+^ atoms and 38 water molecules in the asymmetric unit. The structure was refined to a final R_work_ and R_free_ of 18.7 and 22.3%, respectively. The Ramachandran plot outliers of SeMet-RstA RD are mostly located on the C-terminus (Arg117 of chain A and Leu116 of chain B) and in loop regions (Asp9, Asp10 on chain B and Ile53 on chain C). Detailed refinement parameters for both structures are available in Supplemental Table S2. Coordinates of *kp*RstA DBD/DNA and *kp*RstA RD have been deposited to the PDB with accession codes: 4NHJ and 4NIC, respectively.

## RESULTS

### Promoter binding affinity and thermodynamics of kpRstA–RstA box interaction

The binding affinities of *kp*RstA DBD to the upstream half-site of the RstA box (DNA-16a), downstream half-site of the RstA box (DNA-16b) and the full-length RstA box (DNA-22) were studied by isothermal titration calorimetry (Figure 1). As shown on Figure 1B and Table 1, the titration curve of *kp*RstA DBD to DNA-16a could be fitted well with a single binding site of *K*_D_ = 8.17 ± 0.54 μM with Δ*H* = −10.12 ± 0.12 kcal/mol and Δ*S* = −10.7 cal/mol/deg, indicating that the binding is enthalpically driven. In contrast, the binding of *kp*RstA DBD to DNA-16b did not generate enough heat to obtain reliable thermodynamic parameters, suggesting extremely weak protein–DNA interactions (Figure 1C). The titration curve of *kp*RstA DBD to the full length RstA box (DNA-22) was fitted with a sequential binding model of *K*_D1_ = 10.3 ± 1.13 μM, Δ*H*_1_ = −9.48 ± 0.23 kcal/mol, Δ*S*_1_ = −8.98 cal/mol/deg and *K*_D2_ = 5.15 ± 0.26 μM, Δ*H*_2_ = −6.89 ± 0.25 kcal/mol, Δ*S*_2_ = 1.07 cal/mol/deg (Figure 1D). Although the data can be fit equally well with two independent equivalent sites of *K*_D_ = 3.40 ± 0.33 μM, Δ*H* = −7.95 ± 0.08 kcal/mol, Δ*S* = −1.63 cal/mol/deg, the results would not agree with the half-site binding results which showed that the downstream half-site binds to DBD poorly. The results suggest that binding of *kp*RstA DBD to the first site enhances the binding of the second *kp*RstA DBD to the second site. Mutation of Arg207, one of the key residues responsible for DNA binding (see Figure 4C), to Ala completely abolished the DNA binding of *kp*RstA DBD (Figure 1E).

**Figure 3.**
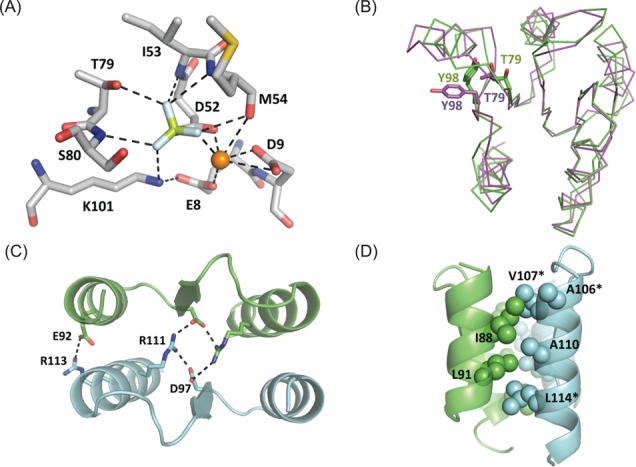
(**A**) Interactions between beryllium fluoride and *kp*RstA RD phosphorylation site. (**B**) Structural alignment of kpRstA RD in the presence of BeF_3_^−^ (green) and inactivated PhoP RD (magenta) shows significant conformational changes of the switch residues Thr79 and Tyr98. (**C**) The intermolecular interface is stabilized by two salt bridges formed between Asp97 (β5)–Arg111 (α5) and Glu92 (α4)–Arg113 (α5). Salt bridges are shown as dotted lines. (**D**) The α4 and α5 helices are packed together through a hydrophobic patch (spheres) formed by Ile88, Leu91, Ala106, Val107, Ala110 and Leu114. Stars (*) denote non-conserved residues among the OmpR/PhoB subfamily.

**Figure 4.**
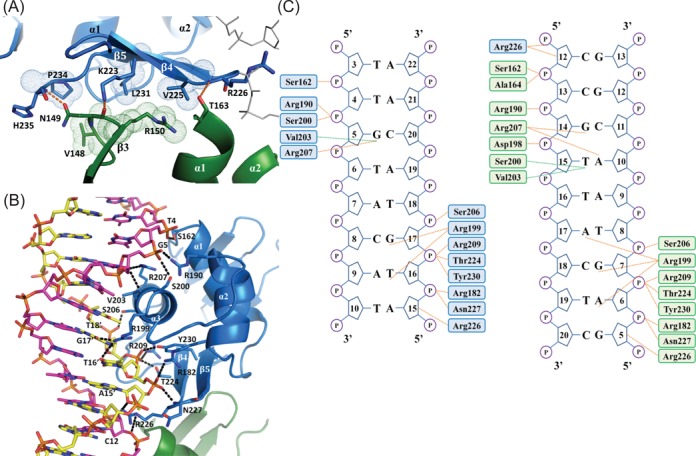
Intermolecular interactions in the *kp*RstA DBD/DNA-23 complex. (**A**) Protein–protein interactions between the upstream (blue) and downstream (green) DBDs. Hydrogen bonds and hydrophobic interactions are shown in orange dashes and space-fill dots, respectively. (**B**) Interactions between the upstream DBD protomer and DNA-23. The coding and template strands are colored magenta and yellow, respectively. (**C**) Schematic of the interactions between the DBDs and DNA-23. The upstream and downstream DBDs are represented by blue and green colors, respectively. Orange dots represent hydrogen bonds and salt bridges, and green dots represent van der Waals interactions. Residues involved in nonspecific interactions are located at helices α1 (Ser162), α2 (Arg182, Arg190), α3 (Ser200, Ser206, Arg209), the C-terminal β-hairpin (Thr224, Asn226, Asn227) and β5 (Tyr230).

**Table 1.** Binding parameters of full-length (FL) *kp*RstA and *kp*RstA DBD towards RstA box DNA sequences

Sample	Model	*N*	*K*_D_ (μM)	Δ*H* (cal/mol)	Δ*S* (cal/mol/deg)
DBD/DNA-16a	One-site	1.05	8.17 ± 0.53	−10100 ± 120	−10.7
					
DBD/DNA-16b	-	-	-	-	-
					
DBD/DNA-22	Sequential	Set as 2	10.31 ± 1.14	−9480 ± 233	−8.98
			5.15 ± 0.3	−6893 ± 252	1.07
DBD-R207A/DNA-22	-	-	-	-	-
					
FL/DNA-22 $\left( {{\rm without}\;{\rm BeF}_3^ - } \right)$	Sequential	Set as 2	45.45 ± 8.41	−9792 ± 1070	−13.0
			5.05 ± 0.72	−478.7 ± 1110	22.6
					
FL/DNA-22 $\left( {{\rm with}\;{\rm BeF}_3^ - } \right)$	Sequential	Set as 2	9.14 ± 1.84	−17400 ± 1380	−35.3
			0.59 ± 0.09	−3028 ± 1400	18.4

The titration curve of full-length *kp*RstA protein to DNA-22 was characterized by a biphasic behavior. The binding isotherms fit poorly to either a one-site model or multiple-site model and only the sequential binding model gave a satisfactory fit with *K*_D1_ = 45.45 ± 8.41 μM, Δ*H*_1_ = −9.79 ± 1.07 kcal/mol, Δ*S*_1_ = −13.0 cal/mol/deg and *K*_D2_ = 5.05 ± 0.72 μM, Δ*H*_2_ = −0.48 ± 1.11 kcal/mol, Δ*S*_2_ = 22.6 cal/mol/deg (Figure 1F). The lower affinity of the first site compared to that of *kp*RstA DBD is consistent with the inhibitory effect of the RD on DBD binding to DNA reported for other TCS response regulators. The RD of PrrA, e.g. sterically blocks the DNA-binding site of its DBD in the absence of BeF_3_^−^, which results in lower affinity of full-length PrrA towards DNA when compared to the DBD by itself (48). PhoB exemplifies another possibility, where the RD is capable of adopting an alternative dimer conformation in the absence of BeF_3_^−^ that has been proposed to interfere with the correct alignment of the DBDs for binding (49,50). Interestingly, binding to the second site was entropy-driven, which suggests changes in conformation and/or formation of hydrophobic contacts. The binding affinity of the full-length *kp*RstA towards DNA-22 was significantly enhanced in the presence of BeF_3_^−^ (*K*_D1_ = 9.14 ± 1.84 μM and *K*_D2_ = 0.59 ± 0.09 μM), primarily through increases in binding enthalpy, although strong entropic contributions persisted for the second site (see Table 1). Size exclusion chromatography showed that the presence of BeF_3_^−^ induced dimerization of full-length *kp*RstA (data not shown), which suggests that the enhanced binding is caused by the dimerization of the protein.

### Structural overview of kpRstA RD, DBD and DBD/DNA complex

The structure of *kp*RstA RD in the presence of BeF_3_^−^ was solved to a resolution of 3.2 Å (Figure 2A). Similar to the structure reported for TorR RD (51), four *kp*RstA RD molecules, which correspond to two dimers, were found in the asymmetric unit (see Supplemental Figure S2). The two dimers within the asymmetric unit had similar structures as revealed by structural alignment, with an rmsd of 0.47 Å over 117 Cα. Structural analyses revealed that the overall structure of *kp*RstA RD has the traditional (βα)_5_ fold of RR regulatory domains that consists of a central five-stranded parallel β-sheet surrounded by five-helices.

The structural topology of *kp*RstA DBD in solution consists of the N-terminal three-strand antiparallel β-sheet (β1, Thr137–Ser139; β2, Leu143–Asp146; β3, Arg149–Leu153) followed by three helices assuming the helix-turn-helix DNA binding motif topology (α1, Thr63–Thr174; α2, Arg182–Arg190; α3, Asp198–Leu212) and ending with a β-hairpin (β4, Ile222–Val225; β5, Lys228–Phe232) (Figure 2B). The N-terminal β-sheet interacts with helix α1 primarily through hydrophobic interactions while stacking interaction between the aromatic rings of Phe140 and Phe232 brings the N-terminal β-sheet to the vicinity of the C-terminus. The overall structure of *kp*RstA DBD shares a winged helix-turn-helix (wHTH) fold typical for the OmpR/PhoB RR superfamily, such as OmpR (PDB ID: 1OPC), PhoB (PDB ID: 1GXQ/1GXP) and KdpE (PDB ID: 3ZQ7), with Cα rmsd ranging from 1.1 to 1.4 Å.

The crystal structure of *kp*RstA DBD/DNA-23 consists of two protein molecules and one 23-bp DNA per asymmetric unit (Figure 2C). In the crystal packing, the DNA duplexes formed a pseudo-continuous DNA helix stabilized by base-stacking interactions between symmetry-related DNA. Superimposition of the two *kp*RstA DBDs in the asymmetric unit showed that their structures are very similar, with an rmsd of 0.67 Å over 93 Cα atoms (see Suplemental Figure S2). The two DBDs bind on the same side of the DNA in a head-to-tail fashion and the electrostatic potential surface of the protein dimer forms a long and continuous positively charged region for DNA binding. The α3 recognition helix is docked into the major groove of the DNA and forms numerous interactions with the RstA box. The C-terminal β-hairpin also makes contact with the DNA, mostly close to the phosphate backbone. Unlike the bent DNA conformation observed for the PhoB DBD/DNA complex (21), the DNA-23 in *kp*RstA DBD/DNA-23 complex only displayed slight bending (∼10°).

### Dimerization of kpRstA RD

All structures of the OmpR/PhoB superfamily RRs solved to date adopt a similar dimeric structure in the phosphorylated (active) state, which can be mimicked by the BeF_3_^−^-bound form (6). In *kp*RstA RD, the BeF_3_^−^ molecule is non-covalently bound to Asp52 within the phosphorylation site, and forms a hydrogen bond with the side-chain of Thr79, a salt bridge to Lys101, and van der Waals contacts with the backbone nitrogen atoms of Ile53, Met54 and Ser80 (Figure 3A). One of the fluorine atoms is coordinated to an Mg^2+^ atom, which in turn coordinates with the main chain carboxyl oxygen of Met54 and the side-chain carboxyl oxygen atoms of Glu8, Asp9 and Asp52 to form a 6-fold coordination of octahedral geometry. The interactions found in the phosphorylation site of *kp*RstA RD are analogous to those of other structures of active RRs from different subfamilies, underscoring the conserved nature of the phosphorylation site environment (6).

Previous studies on receiver domains of RRs have demonstrated that a molecular switch comprised of a highly conserved residue pair (Thr/Ser and Tyr/Phe) was able to exchange between inward (active) and outward (inactive) conformations in response to phosphorylation (51–54). Superimposing the structure of activated *kp*RstA RD onto the inactive PhoP RD (PDB ID: 2PKX) suggests that the signature switch residues are likely Thr79 and Tyr98, which are located in the β4–α4 loop and β5, respectively (Figure 3B) (53). This molecular switch could propagate the structural changes at the phosphorylation site to the dimer interface, which spans the α4–β5–α5 region. Residues in this region are highly conserved in the OmpR/PhoB superfamily of RRs. The dimer interface of *kp*RstA RD has a buried surface area of 689.5 Å^2^ as calculated with PISA (55). Two conserved intermolecular salt bridges are formed between Asp97 (β5)–Arg111 (α5) at the center and Glu92 (α4)–Arg113 (α5) at the outer sides of the interface (Figure 3C). Additionally, six hydrophobic residues (Ile88, Leu91, Ala106, Val107, Ala110 and Leu114) cluster together to form an extensive hydrophobic patch that holds together the α4 and α5 helices between adjacent molecules (Figure 3D).

### Interaction between kpRstA DBD and DNA

The two DNA-bound DBDs interact with each other with a buried surface area of 362 Å^2^ per monomer at the protein–protein interface. The interface mostly consists of β4 and β5 of the upstream DBD (bound to the 5′ half-site of the coding strand) on one end and the two loops between β2–β3 and β3–α1 of the downstream DBD (bound to the 3′ half-site of the coding strand) on the other end (Figure 4A). Intermolecular van der Waals contacts are formed between Gly177 (α1–α2 loop)–Val148 (β2–β3 loop), Glu218 (α3–β4 loop)–Ala160 (β3–α1 loop), Val225 (β4)–Arg150 (β3), Val225 (β4)–Thr163 (α1), Leu231 (β4)–Arg150 (β3), Pro234 (C-terminal tail)–Val148 (β2–β3 loop). Moreover, Lys223 (β4) and His235 (C-terminal tail) form hydrogen bonds with Asn149 (β2–β3 loop) and Arg226 (β4–β5 loop) forms a hydrogen bond with Thr163 (α1). Lys223 (β4) is also within hydrogen bonding distance to Arg150 (β3). The extensive interactions observed between the upstream and downstream DBDs suggest that the two binding events are not independent of each other, and formed the basis for the choice of a sequential binding model when fitting the ITC data. Similar DBD–DBD interfaces have been observed for DNA-bound PhoB and KdpE (21,56), even though the residues involved share little homology amongst the different proteins (see Supplemental Figure S1).

The two *kp*RstA DBDs within an asymmetric unit bind to the DNA with similar contacts (Figure 4B and C), with contact surfaces of 882 Å^2^ between DNA and protein and 899 Å^2^ for the upstream and downstream protomers, respectively. The upstream DBD binds the RstA box from T4 (coding strand) to A15′ (the prime sign indicates the template strand), and the downstream DBD binds from C13 to G5′ (Figure 4B). Specific interactions between the α3 recognition helix residues and DNA, which include Val203-T6, Arg207-G5, Arg199-G17′ and Arg199-T16′, provide the basis for sequence specificity. It should be noted that while the upstream Arg199 forms two specific hydrogen bonds with the template strand of the RstA box TACA, G17′ and T16′, the downstream Arg199 contacts the DNA duplex by forming three hydrogen bonds: Arg199-A17, Arg199-G17′ and Arg199-A6′ (Figure 4C). This observation implies that the side chain of Arg199 is capable of recognizing nucleotide sequences that deviate from the canonical RstA box. Similarly, there are numerous interactions between the second *kp*RstA DBD and the second RstA box. Base specificity is established mainly through the residues Arg200, Ser201, Val204 and Arg208 (Figure 4C).

**Figure 5.**
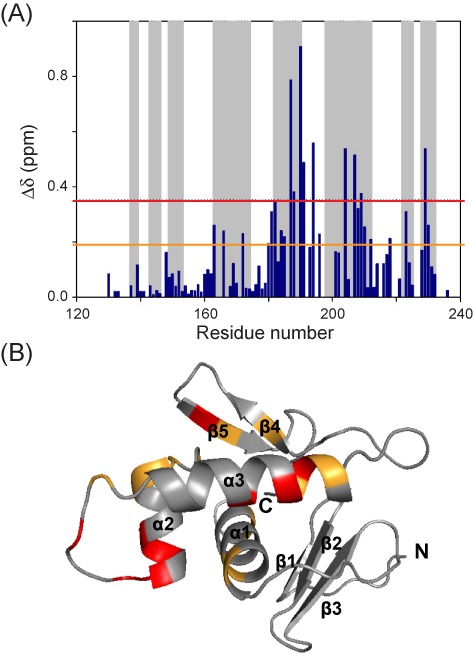
Interaction between *kp*RstA DBD and DNA-16a. The normalized chemical shift perturbations (CSP) of *kp*RstA DBD induced by binding to DNA are shown in (**A**). Residues with CSP above the upper (red) and lower (orange) lines are mapped to the DBD structure in (**B**).

The binding interface of the *kp*RstA DBD/DNA complex in solution was also examined through NMR CSP studies (Figure 5A and B). Residues perturbed by the binding of DNA-16a localize primarily at the C-terminal region including helices α2, α3, β4, β5 and the α2–α3 loop, with the largest perturbations occurring at or near the α3 recognition helix. Slight perturbation of the N-terminal region of α1 was also observed. Noticeably, the N-terminal β-sheet showed very little perturbation. The observed CSPs are in full agreement with the DBD/DNA interactions identified in the crystal structure of the complex.

### Structural and backbone dynamics changes of kpRstA DBD upon DNA binding

**Figure 6.**
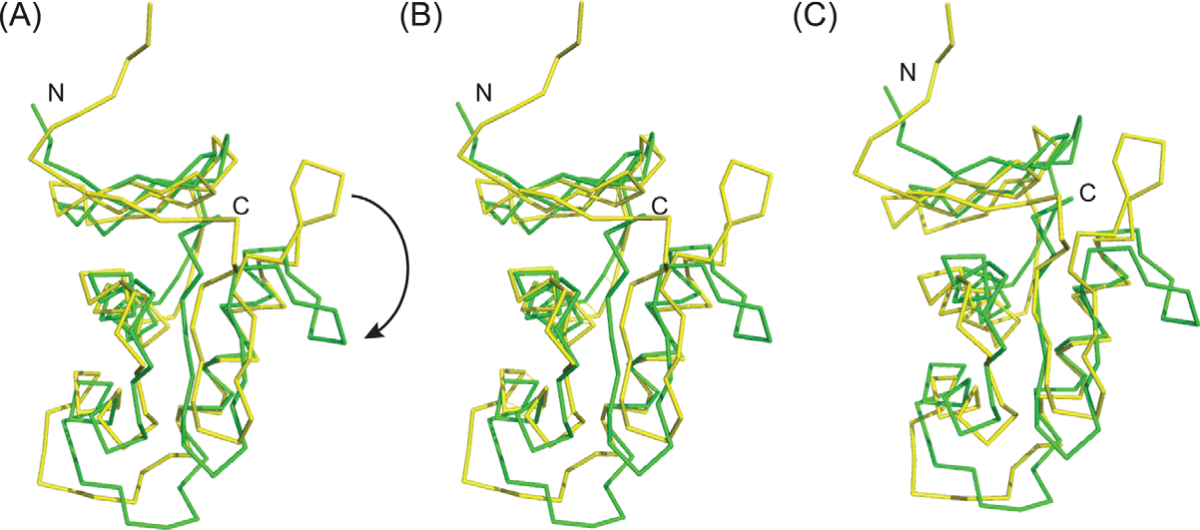
Conformational changes of *kp*RstA DBD upon binding to the RstA box DNA. (**A**) Superposition of the DNA-free (yellow) and DNA-bound (green) structures of the DBD. The apex of the α3–β4 loop swings down towards the α3 helix (shown in arrow). (**B**) Superposition of the same structures as (A) by superimposing the N-terminal region (res. 135–190). (**C**) Superposition of the same structures as (A) by superimposing the C-terminal region (res. 191–235).

Superposition of the DNA-free NMR structure and DNA-bound crystal structure of the DBD revealed that their overall structures are similar with an rmsd of 1.43 Å for 101 Cα atoms (Figure 6A). However, superimposing the N-terminal halves (res. 135–190) of the two structures resulted in an rmsd of 1.12 Å for 52 Cα atoms (Figure 6B), whereas superposition of the C-terminal halves (res. 191–235) resulted in an rmsd of 1.70 Å for 36 Cα atoms (Figure 6C), indicating that the region spanning the transactivation loop to β4 and β5 undergoes large conformational changes upon DNA-binding. Noticeably, the α3–β4 loop swings ∼90° towards the template strand of the DNA in the complex, which results in Ala216 interacting with the ribose part of G17′. Moreover, insertion of Arg226 into the minor groove brings the β4–β5 loop into contact with the DNA and clamps down the template strand alongside the α3 recognition helix, further stabilizing the interaction between the DBD and DNA.

**Figure 7.**
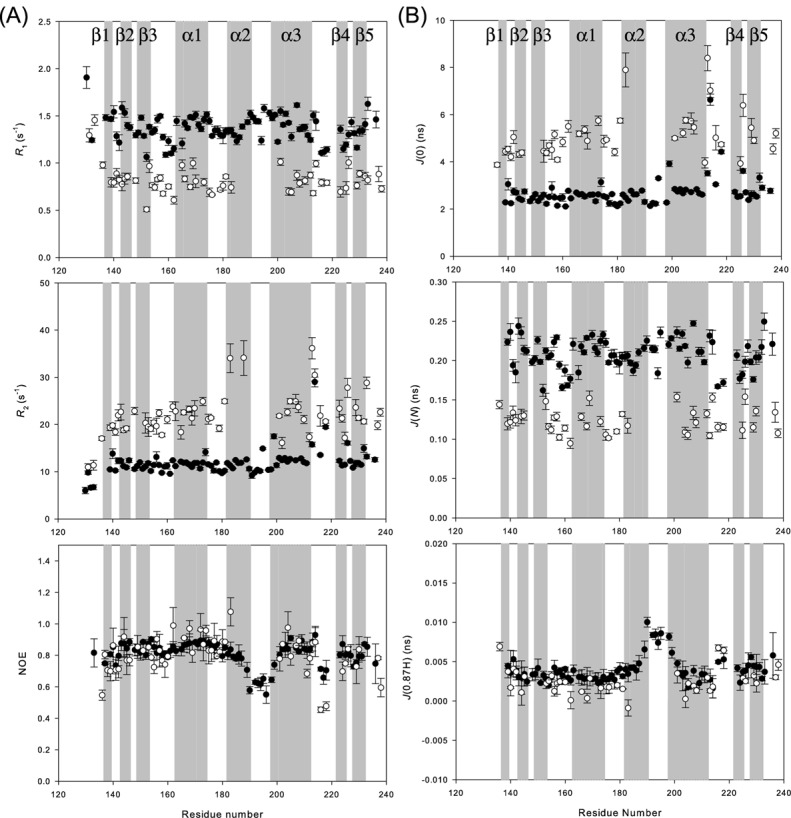
Dynamics of *kp*RstA DBD as probed by NMR. (**A**) Sequence variations of the ^1^H-, ^15^N-R_1_ (top), ^15^N-R_2_ (middle) and ^15^N NOE (bottom) of *kp*RstA DBD (filled circles) and *kp*RstA DBD/DNA-16a complex (open circles). (**B**) Sequence variations of the reduced spectral density functions *J*(0) (top), *J*(N) (middle) and *J*(0.87H) (bottom) deduced from the dynamic data shown in (A).

To gain further insights into the dynamic behavior of *kp*RstA DBD, we investigated the backbone dynamics of DNA-free *kp*RstA DBD and *kp*RstA DBD/DNA-16a complex by measuring ^1^^5^N-R_1_, ^15^N-R_2_ and [^1^H–^15^N]-NOE at 800 MHz (Figure 7A) and calculating the reduced spectral density functions from these relaxation parameters (33,34,57,58). Reduced spectral density functions reflect the degree of motion in particular frequency regions. Thus, higher value of *J*(*ω*) indicates the presence of motion at the frequency ω MHz region. The results are shown in Figure 7B. *J*(0), *J*(N) and *J*(0.87H) represent reduced spectral density function at 0, 80 and 696 MHz, respectively. Overall, the DNA-free *kp*RstA DBD (filled circles) is rigid, except two loop regions. The loop connecting α2 and α3 (Lys187–Ser200) is characterized by large *J*(0.87H) indicative of the presence of high frequency fast motion. Another loop region showing flexibility is that connecting α3 and β4 (Ala216–Glu218). This region also showed elevated *J*(0.87H) suggesting the presence of fast motion. Moreover, this loop region also showed considerable slow motion, as indicated by large *J*(0). Interestingly, these two loop regions juxtapose helix α3. In the DNA-bound form (open circles), the DBD showed a similar dynamic behavior and the two flexible loops between Ala216–Glu218 and Lys187–Ser200 also exhibit higher flexibility as observed in the free form. However, reliable relaxation measurements cannot be obtained for many resonances between α2 and α3, indicating that this region becomes more flexible upon DNA-binding. One also observes higher and more scattered *J*(0) for the α3–β4 region, suggesting that the entire C-terminal region beyond α2 exhibit slow motion, possibly some conformational exchange process. The functional significance of the enhanced dynamics of the two regions is discussed in the following section. In agreement with the monomeric protein sizes, the overall rotational correlation times deduced from the relaxation data are 6.75 and 12.5 ns for the DNA-free and DNA-bound form, respectively.

## DISCUSSION

### *kp*RstA DBD binds DNA in sequential mode

Modulation of gene expression by RRs in bacteria has been well studied, and much of the structural changes resulting from the activation of the RD are relatively well understood (51,52,54,59). On the other hand, the DNA-bound structure of PhoB DBD has for a long time been the only source of information for structural changes of the DBD upon binding to DNA (21). Recently, the structures of *K. pneumoniae* PmrA DBD and *E. coli* KdpE bound to their respective cognate DNA sequences have been solved (22,56). Although PhoB, PmrA and KdpE belong to the same OmpR/PhoB subfamily, their DBDs have shown very different structural and functional characteristics when bound to DNA. For example, binding of PhoB to its cognate sequence introduces a ∼40° bend in the DNA, whereas binding of KdpE or PmrA to DNA only introduces slight bending. However, binding of KdpE to DNA results in a much larger dimer surface than in PmrA. This lack of a consensus binding mechanism is also observed for *kp*RstA, which belongs to the same subfamily. *kp*RstA DBD binds to the RstA box without inducing substantial bending of the DNA and forms a dimer interface comparable to that of KdpE. Yet unlike KdpE, binding of *kp*RstA DBD to the two half-sites of the RstA box have very different affinities, whereas KdpE DBD reportedly binds to its two cognate half-sites with similar affinity (60). Moreover, binding of full-length *kp*RstA to the full-length RstA box is clearly biphasic in sequential mode (Figure 1D and E), which has never been reported for other RRs from the OmpR/PhoB subfamily. These observations suggest that the DNA-binding properties of RRs in the same family need to be examined on an individual basis, even though they might share a similar structural fold.

The affinities of *kp*RstA DBD and full-length *kp*RstA toward cognate DNA measured in this study are generally weaker than those reported for other RRs. PmrA and KdpE DBDs all bind to their respective high-affinity half-sites at sub-micromolar concentrations (22,60), which are one to two orders of magnitude stronger than the affinity of *kp*RstA DBD towards DNA-16a (Figure 2A). Full-length *kp*RstA also binds to the full-length RstA box (Figure 2E and F) at lower affinity than binding of PhoB towards a consensus *pho* box (61). Although all response regulators with data available show no difference in either structure or function between the BeF_3_^−^-bound and phosphorylated forms (62), we cannot exclude the possibility that BeF_3_^−^ is not able to activate *kp*RstA to the same extent as phosphorylation. Interestingly, the promoter region of the *asr* operon harbors binding sites to both RstA and PhoB, although either protein is capable of activating the operon by itself (7). We speculate that the arrangement of a strong- and weak-binding element would enhance the dynamic range of gene expression and allow for fine-tuning of gene regulation. The *csgD* promoter region also contains RstA and OmpR binding sites, but in this case the two sites overlap extensively (7). Since the RstB/RstA and EnvZ/OmpR TCS respond to different types of environmental stress, the large difference in DNA-binding affinity between RstA and OmpR should have little relevance on the regulation of CsgD by RstA.

The lack of affinity for the downstream half-site displayed by the DBD and the biphasic binding behavior of full-length *kp*RstA indicate that the two DBDs in the *kp*RstA dimer bind to the full-length RstA box in a sequential fashion (Figure 2). Our structural studies suggest that binding of the first DBD to the upstream half-site bring the α3–β4 loop close to the DNA, which would then place Glu218 in a position to form inter-DBD contacts with Ala160 (Figure 4B). Backbone dynamics further suggest that Arg226 becomes more rigid when interacting with DNA, as observed from the increase in *J*(0) and concomitant decrease in *J*(N) and *J*(0.87H) in Figure 7, which would help stabilize the Arg226–Thr163 inter-DBD hydrogen bond. Thus, binding of one *kp*RstA DBD promotes the binding of the second DBD to the second site on RstA box in a cooperative manner. Whether this sequential binding behavior is dependent on the sequence of the DNA, particularly of the downstream half-site is currently unknown. It is worth noting that, unlike in *E. coli*, neither *asr* nor *csgD* of *K. pneumoniae* contain the classic RstA box (TACANNNNNNTACA) sequences in their promoter region. In fact, *K. pneumoniae* homologs of the putative *E. coli* genes that may be targets of RstA as proposed by Osagawara et al. (8) did not harbor the classic RstA box either. The unexpected absence of classic RstA boxes in homologous genes hints at divergent RstA–DNA recognition between *K. pneumoniae* and *E. coli*.

### RD dimerization is entropy driven

Binding of the second DBD to the second half-site features a strong entropic component (see Table 1) contingent on the presence of the RD. In protein–DNA complexes, favorable binding entropy primarily originates from desolvation of nonpolar surfaces (63). Incidentally, the RD of *kp*RstA contains an extensive hydrophobic patch at the α4–β5–α5 region, which forms the dimer interface in the active form (Figure 3D). Inactive *kp*RstA RD spontaneously forms dimers at high concentrations as observed from the line-widths in NMR spectra (data not shown), suggesting that the dimer interface is exposed to the solvent. The α4–β5–α5 region also contains a number of charged residues, which may harbor non-specific binding sites for anions and cations. Based on these premises, we would argue that the entropic contribution arises from the release of water and ions at the α4–β5–α5 region upon RD dimerization, which is induced by the binding of the second DBD to the downstream half-site. However, in the presence of BeF_3_^−^, one would expect the dimerization to occur before titrating the protein into the DNA solution, hence the entropic energy should noticeably decrease. This is not observed in our ITC experiments. One possibility is that the RD dimer interface within the full-length *kp*RstA can access different conformations depending on whether the DBD is bound to DNA and the RD binds BeF_3_^−^, and only when both types of interactions are present will the interface adopt its final form, similar to the multiple states observed for the CheY response regulator under different experimental conditions (64,65). Effort is currently under way to test this possibility.

### The ‘prime-and-lock’ model for the activation of *kp*RstA

**Figure 8.**
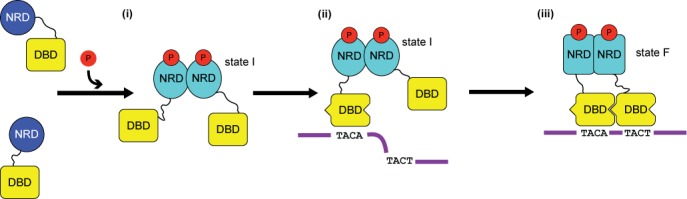
Proposed structure-based model for binding of *kp*RstA to the RstA box DNA. (i) phosphorylation of the RD results in formation of *kp*RstA dimers with an intermediate RD–RD interface conformation (state I). Due to the presence of two DBDs close in space, the DNA-binding affinity is increased compared to the unphosphorylated form. (ii) Binding of the first DBD to the upstream half-site of the RstA box causes conformational changes in the first DBD, ‘priming’ it for interaction with the second DBD. (iii) The second DBD binds to the downstream half-site and ‘locks’ the complex through DBD–DBD and RD–RD interactions. At the same time, the RD–RD dimer assumes its final conformation (state F), providing the entropic energy required for the second binding event.

Taken together, we propose that binding between *kp*RstA and its cognate DNA sequences utilizes a ‘prime-and-lock’ mechanism, shown in Figure 8. The process is divided into the following steps: (i) phosphorylation of the RD results in formation of RstA dimers with an intermediate RD–RD interface conformation and concomitant release of the inhibitory effect by the RD (state I). (ii) Binding of the first DBD on the upstream half-site of the RstA box causes conformational changes in the first DBD, ‘priming’ it for interaction with the second DBD. The transactivation loop (Gly191–Arg199) also becomes more flexible (see Figure 7). (iii) The second DBD binds to the downstream half-site and ‘locks’ the complex through DBD–DBD and RD–RD interactions. At the same time, the RD–RD dimer assumes its final conformation (state F), providing the entropic energy required for the second binding event. The flexible transactivation loop is now in position to recruit the σ subunit of RNA polymerase for downstream gene activation (66). The enhanced dynamics allows the transactivation loop to sample the space around the promoter in a short time, increasing the chance of encountering the σ subunit. Thus, our model presents the following highlights: (a) Phosphorylation of the RD is a pre-requisite for *kp*RstA to bind to DNA, since otherwise the binding affinity would be too weak. As such, tight control of RstA-activated genes can be achieved simply by controlling the population of phosphorylated *kp*RstA. (b) Binding of both DBDs within a *kp*RstA dimer to the DNA is necessary for the formation of a stable activation complex. The first binding event anchors the dimer to potential binding sites, allowing the second binding event to occur provided that a downstream half-site is available. In the absence of the downstream half-site (i.e. wrong binding site), the binding affinity of the first DBD towards the upstream half-site alone would not be enough to maintain the activation complex. (c) Binding to DNA instigates functionally relevant changes in backbone dynamics of the DBD. The current paradigm for regulation in RRs emphasizes the importance of interfaces created between domains rather than conformational changes (56,67). Also, the importance of interfaces in promoting the strong binding of RstA to DNA is clearly the most important factor in cooperative binding of the second DBD to DNA. We observed extensive interactions between the upstream and downstream DBDs indicating that the two binding events are not independent of each other, and formed the basis for the choice of a sequential binding for our model. However, significant structural changes were also observed between the DNA-free and the DNA-bound RstA DBDs, Noticeably, the α3–β4 transactivation loop swings ∼90° toward the template strand of the DNA in forming the complex. In conclusion, our model provides a framework for RstA–DNA interaction that can be experimentally validated in future studies.

## ACCESSION NUMBERS

PDB IDs: 2MLK, 1ZES, 4NHJ, 4NIC, 1OPC, 1GXQ, 1GXP, 3ZQ7 and 2PKX.

## SUPPLEMENTARY DATA

Supplementary Data are available at NAR Online.

SUPPLEMENTARY DATA
