# Supplementary material for: Structural dynamics of the two-component response regulator RstA in recognition of promoter DNA element
Source: Nucleic Acids Res. 2014 Jul 2;42(13):8777–88. doi: 10.1093/nar/gku572 (PMC4117788; doi:10.1093/nar/gku572)
Supplement: SUPPLEMENTARY DATA [file supp_42_13_8777__index.html]

Structural dynamics of the two-component response regulator RstA in recognition of promoter DNA element — SUPPLEMENTARY DATA 

# Structural dynamics of the two-component response regulator RstA in recognition of promoter DNA element

## SUPPLEMENTARY DATA

**Files in this Data Supplement:**

- Supplementary Table
