# Supplementary material for: Structural dynamics of the two-component response regulator RstA in recognition of promoter DNA element
Source: Nucleic Acids Res. 2014 Jul 2;42(13):8777–88. doi: 10.1093/nar/gku572 (PMC4117788; doi:10.1093/nar/gku572)

Supplementary Data

Table S1. Mutagenesis primers for kpRstA DBD.

L153M_sense	5′-CCGGTCAACCGCCAGGTGATGCTTGGCGGAGAAAAC-3′
L153M_antisense	5′-CACCTGGCGGTTGACCGGGTCAATGGTCAGCGA-3′
L168M_sense	5′-CTGTCGACCGCCGATTTCGATATGCTGTGGGAGTTGGCC-3′
L168M_antisense	5′-ATCGAAATCGGCGGTCGACAGGGCGACGTTTTCTCC-3′
R207A_sense	5′-CGACGTGGCTATCTCCGGGGTGCGCAAAAAACTG-3′
R207A_antisense	5′-CAGTTTTTTGCGCAGGGCGGAGATAGCCACGTCG-3′

Table S2. Structural data and refinement statistics

**Crystal**	RstA DBD/DNA complex	SeMet-RstA DBD/DNA complex			SeMet-RstA RD	
	Native	High	Edge	Peak	Edge	Peak
Wavelength (Å)	0.97862	0.96357	0.97879	0.97862	0.97891	0.97874
Space group	$P2_12_12$				$P6_522$	
Cell dimensions						
a (Å)	159.9		158.2		113.2	113.5
b (Å)	33.3		33.2		113.2	113.5
c (Å)	72.8		72.7		242.9	243.7
Resolution (Å)	50.0 – 2.7	50.0 – 3.2	50.0 – 3.3	50.0 – 3.3	30.0 – 3.1	30.0 – 3.2
Redundancy	10.0 (10.1)	4.2 (3.9)	4.2 (3.8)	4.2 (4.0)	6.0 (6.6)	9.1 (10.0)
Completeness (%)	92.0 (82.9)	99.0 (98.8)	97.6 (97.2)	99.1 (98.3)	98.0 (99.8)	98.8 (99.9)
I/σ (I)	60.0 (7.2)	36.9 (20.5)	37.5 (20.9)	38.7 (19.7)	15.7 (6.8)	23.6 (8.2)
R_{merge} (%)[b]	5.9 (27.1)	4.3 (8.4)	4.7 (9.3)	4.7 (7.3)	11.6 (35.4)	9.6 (34.3)
**Refinement**						
Resolution (Å)	25.0 – 2.7				26.6 – 3.2	
R-factor[c]/R_{free} (%)[d]	21.60 / 27.13				18.70 / 22.27	
Number of atoms						
Protein	1601				3688	
Nucleic acid	918					
Water	63				38	
RMSD						
Bonds ($Å^2$)	0.004				0.010	
Angles (°)	0.948				1.369	
B-factor ($Å^2$)						
All atoms	63.71				58.04	
Protein	65.74				58.03	
Nucleic acid	60.13					
Water	57.27				59.63	
Ramachandran statistics:						
Favored (%)	93.97				93.48	
Allowed (%)	6.03				5.65	
Outlier (%)	0.00				0.87	
**Solution**	RstA DBD					

Number of experimental NMR restraints:	
Intraresidue & Interresidue sequential, $\|i-j\|<=1$	510
Interresidue medium-range, $1<\|i-j\|<5$	154
Interresidue long-range, $\|i-j\|>=5$	216
Dihedral ϕ and ψ angle constraints from TALOS	153
CYANA structural statistics:	
RMSD to mean structure* (Å):	
Backbone	0.82 ± 0.15
Heavy atom	1.47 ± 0.15
RMSD to mean structure (secondary structure regions)# (Å):	
Backbone	0.52 ± 0.10
Heavy atom	1.10 ± 0.09
	3.33
Ramachandran statistics:	
Most favored regions	77.8%
Additionally allowed regions	22.1%
Generously allowed regions	0.1%
Disallowed regions	0%

a. Values in the parenthesis are for the highest resolution shell

b. $R_{merge} = \Sigma \mid I - \langle I \rangle \mid / \Sigma I$, where I = observed intensity, and <I> = average intensity from multiple observations of symmetry related reflections.

c. R = P | Fobs- Fcalc | /PFobs, where Fobs and Fcalc are observed and calculated structure factor amplitudes.

d. Rfree was calculated on the basis of 10 % of the total number of reflections randomly omitted from the refinement.

* Except for Met121-His135 and Trp237-Asn239.

\# Calculated for residues 138-140, 143-146, 151-154, 157-158, 163-174, 182-189, 198-211, 223-224, and 230-231.

Figure S1. Sequence comparison of *kp*RstA with other response regulators from the OmpR/PhoB subfamily.

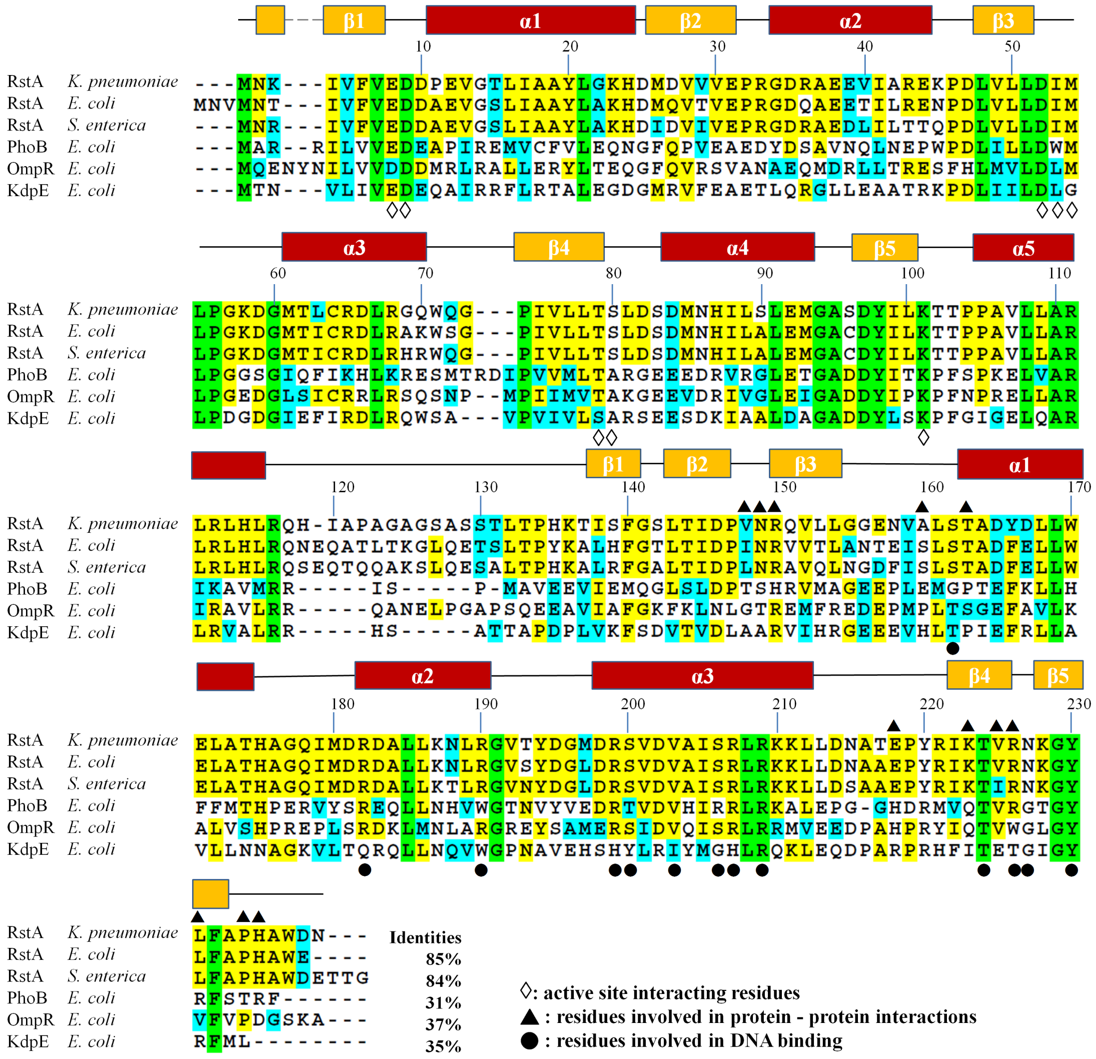

Figure S2. (A) Tetramer assembly of kpRstA NRD in an asymmetric unit. (B) Superposition of the upstream (blue) and downstream (green) protomers of the DBD in the DBD/DNA complex.

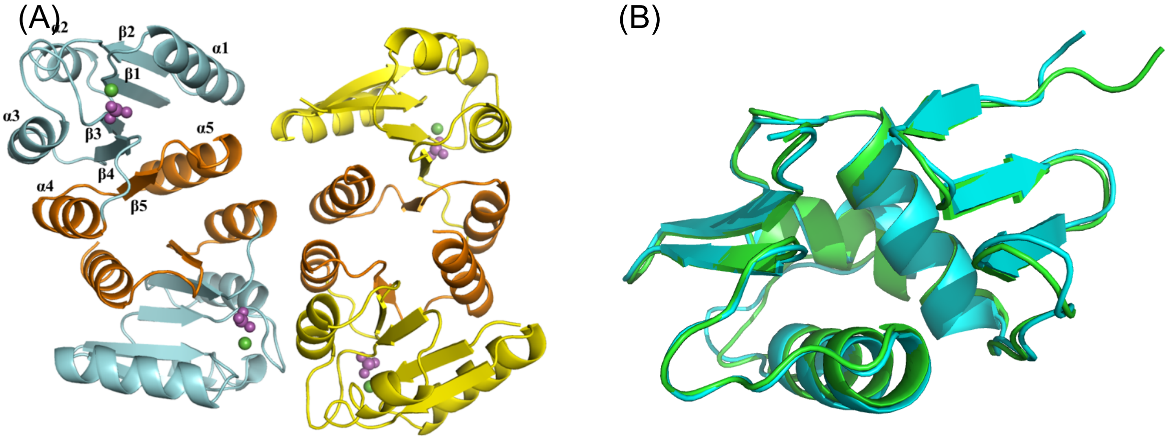

Supplement: SUPPLEMENTARY DATA [file supp_gku572_nar-01205-h-2014-File011.pdf]
